# New Caledonian Crows Rapidly Solve a Collaborative Problem without Cooperative Cognition

**DOI:** 10.1371/journal.pone.0133253

**Published:** 2015-08-12

**Authors:** Sarah A. Jelbert, Puja J. Singh, Russell D. Gray, Alex H. Taylor

**Affiliations:** 1 School of Psychology, University of Auckland, Private Bag 92019, Auckland, New Zealand; 2 School of Psychology and Neuroscience, University of St Andrews, St Andrews, United Kingdom; University of Sheffield, UNITED KINGDOM

## Abstract

There is growing comparative evidence that the cognitive bases of cooperation are not unique to humans. However, the selective pressures that lead to the evolution of these mechanisms remain unclear. Here we show that while tool-making New Caledonian crows can produce collaborative behavior, they do not understand the causality of cooperation nor show sensitivity to inequity. Instead, the collaborative behavior produced appears to have been underpinned by the transfer of prior experience. These results suggest that a number of possible selective pressures, including tool manufacture and mobbing behaviours, have not led to the evolution of cooperative cognition in this species. They show that causal cognition can evolve in a domain specific manner–understanding the properties and flexible uses of physical tools does not necessarily enable animals to grasp that a conspecific can be used as a social tool.

## Introduction

Human cooperation is based upon two cognitive building blocks. The first is an understanding of the causality of cooperation: that a conspecific can be used as a social tool to achieve a goal that is inaccessible via individual action. The second is a sense of fairness (inequity aversion): cooperators must avoid situations where they put a lot of effort into a cooperative task and receive little reward compared to their partner(s). Whilst these cognitive mechanisms are well-developed in humans [1], we do not fully understand the selective pressures that led to their evolution. However, by examining what different animal species understand about the role of a partner, and how they react to unequal outcomes in cooperative tasks, we may be able to gain insight into how cooperative cognition evolved.

In recent years, cooperative string-pulling tasks have yielded evidence that pairs of animals from a variety of species can perform actions simultaneously to achieve a goal [2–10]. However, evidence for cooperative cognition is frequently harder to find. Notably, both chimpanzees and elephants not only cooperate, but appear to understand how cooperation works [2–4]. Elephants will wait for up to 45 seconds for a partner to arrive before they begin to act on a cooperative task [2]; and chimpanzees not only wait for a partner, but will actively recruit help when needed [3], remembering previous successes to recruit the most effective partner. Their performance stands in contrast to capuchins, hyenas, parrots and rooks who can solve cooperative tasks, but, when their partners are absent or delayed, fail to wait for them to arrive before acting [5–8]. Dogs are also able to successfully wait for a partner, but only over short delays (2 seconds with a conspecific partner) [9], whereas bonobos, with their high levels of social tolerance, can outperform chimpanzees on some cooperative tasks [10]. Other primates, including orangutans [11] and cotton-top tamarins [12], tested in simultaneous handle-pulling tasks, may also recognize the role of a partner, with some individuals appearing to monitor their partner’s behaviour and coordinate their actions in these tasks. Moreover, female orangutans tested in mother-offspring dyads have been observed to use their offspring directly as social tools, by manipulating the young orangutans’ limbs to retrieve food or tools that are only accessible through small gaps, or by passing their offspring tools to obtain out of reach rewards [13]. Recently, comparable abilities have also been demonstrated in fish. Trout will recruit the more effective of two artificial moray eels in an ecologically relevant collaborative hunting task [14]. However, in this highly specific context it is unclear what cognitive mechanisms underpin the trout’s behaviour. Thus, to date, certain primates, elephants, and perhaps dogs, but arguably not several other species, appear to recognize that other agents can act as social tools. That is, they understand that conspecifics can create effects in the world which–when combined with their own actions–can allow them to achieve goals that cannot be achieved alone.

The second key mechanism for cooperative cognition–inequity aversion–has been identified in some non-human animals (see [15] for review). Chimpanzees, capuchins, macaques and possibly bonobos will reject a reward if a partner receives a better reward for the same effort [16–23]. Domestic dogs stop participating if a partner receives an award but they receive none [24]. However, they do not show sensitivity to reward type or degree of effort, suggesting their inequity aversion may have different cognitive underpinnings. Carrion crows and ravens possibly demonstrate inequity aversion; however, the corvids also reject low value rewards when tested individually, suggesting they may respond only to food value [25]. Orangutans, squirrel monkeys, owl monkeys, marmosets and tamarins are not sensitive to inequity [23,26–29], and elephants have not yet been tested.

At present it is difficult to make firm conclusions about the selective pressures that do, and do not lead, to cooperative cognition. It has been suggested that living in complex social groups leads to enhanced social cognition (i.e. the social intelligence hypothesis [30,31]). However, in terms of the ability to understand that conspecifics can be used as social tools, this ability has been most clearly identified in species that are not only highly social [32–35] (although see orangutans [36]), but also show sophisticated tool behaviors in the wild, such as the use of natural materials for termite fishing (chimpanzees), fly swatting (elephants) or scratching (orangutans) [34,37–41]. It is therefore difficult to pinpoint which of these species’ characteristics could have led to the evolution of the understanding that others can be used as social tools. Has interaction with conspecifics led to sophisticated social cognition, or has an understanding of the causal relations behind tool use and manufacture been transferred to the social domain?

In terms of inequity aversion, it has been suggested that inequity aversion evolves in order to foster long-term cooperation between unrelated individuals [1,42,43]. In particular, Brosnan suggests that responding to inequity facilitates partner choice; increasing an individual’s fitness by enabling them to reject partnerships which repeatedly lead to unequal outcomes [43–45]. In support of this, inequity aversion is found in highly-cooperative capuchins, but not in the closely related, less cooperative squirrel monkey [27]; and in cooperative chimpanzees, but not in typically less cooperative orangutans [26]. However, to date, the only species showing sensitivity to inequity are not just frequent cooperators, but are also highly social in the wild. This leaves open the question of whether the combination of high sociality and cooperation is essential for the evolution of inequity aversion, or whether inequity aversion could also evolve in cooperative species with low levels of sociality.

**Fig 1:**
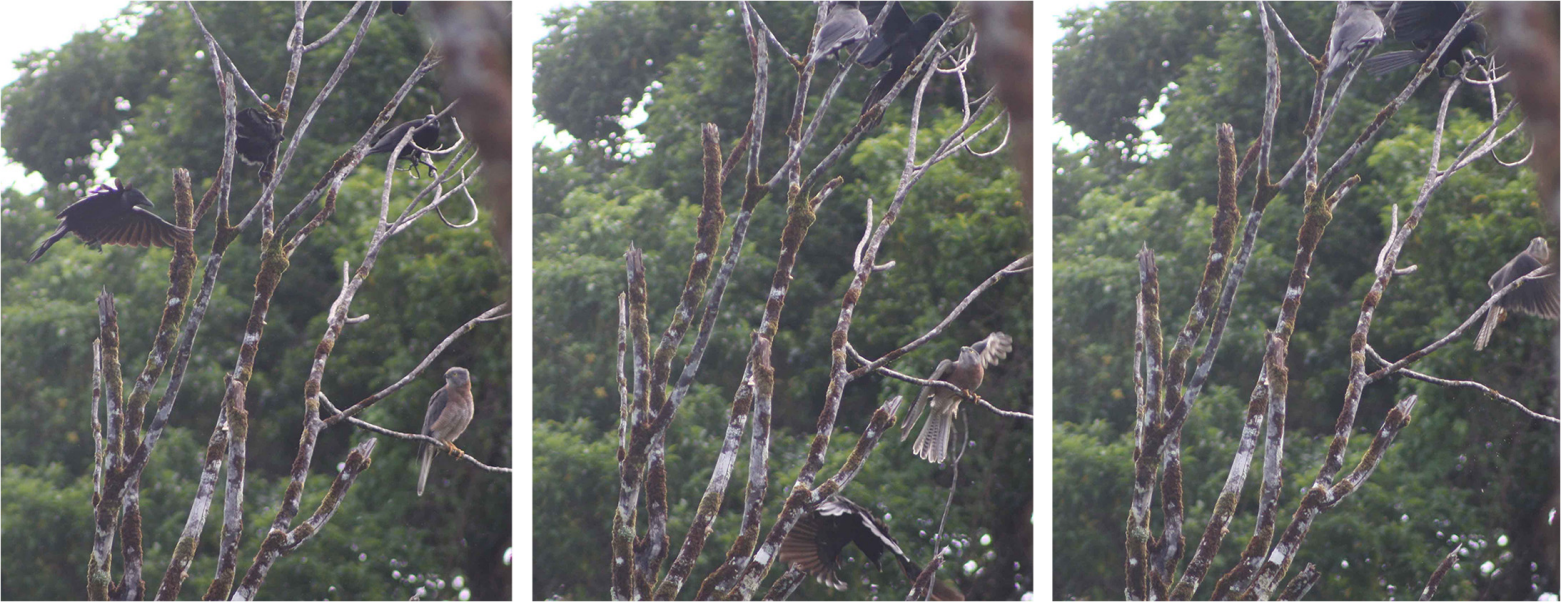
A sequence of photos showing New Caledonian crows mobbing a raptor. Photo credit Mick Sibley.

Here, we investigate the cognitive mechanisms New Caledonian (NC) crows use to tackle problems requiring cooperation. NC crows live in small family groups [46] and appear to mix closely outside of these groups with only 5–6 individuals [47]. These crows therefore have smaller group sizes than both African elephants (mean group size: 6.75 [48,49]) and chimpanzees (mean: 53.5 [50]), as well as other corvid species, such as rooks, which can form groups of over 200 individuals [51]. However, NC crows are known to mob predators such as raptors (see Fig 1); a behaviour which benefits other crows in the area whether they mob or free-ride. Thus, despite their limited sociality, NC crows carry out some cooperative behaviours in the wild, and this characteristic allows us to test whether cooperation, without high levels of sociality, can lead to the evolution of inequity aversion.

Furthermore, NC crows produce a range of complex tool behaviours, both in the wild [52–55] and in captivity [56–59]. They appear to have an abstract understanding of tool use [56,57], and reason about both object-object interactions [60–63] and hidden causal agents [64–67]. Thus, these crows have an understanding of both tool use and physical causal relations that is arguably comparable with chimpanzees’ [60,68,69]. This species is therefore an ideal candidate to test whether social or technical selection pressures shape the evolution of the understanding that a conspecific can be used as a social tool.

In the current study we presented two groups of NC crows with a novel cooperative problem. Crows had to pass a stone to a partner in an adjacent cage, which the partner could use to trigger an apparatus, releasing food into both cages. By manipulating whether or not the partner was present, and the type and amount of food available to each bird, we examined whether NC crows understood the causality of cooperation and were sensitive to inequity in a cooperative task. The first group of crows we tested received a spontaneous stone passing task, followed by a test of inequity aversion. Then, the second group of crows received a spontaneous stone passing task, a social vs. asocial control test, and a different test of inequity aversion. Results are presented for both groups simultaneously.

## Methods

### Ethics Statement

All aspects of this study were conducted under approval from the University of Auckland ethics committee (reference no. R602). The Loyalty Islands Provincial Administration granted us permission to work on Maré, and the Province Sud granted us permission to work on Grande Terre. All birds were caught on private land with permission from the land owners and were released at their site of capture at the end of testing.

### Group 1

#### Subjects

Subjects were 6 wild-caught New Caledonian crows, captured from various sites on the island of Maré between June and September 2010 using Whoosh nets, and housed in a six-cage outdoor aviary. Cages varied in size but all were at least 2m^2^ x 3m. Crows were fed a diet of papaya, meat, dog biscuits and egg, twice daily, and had access to water ad libitum. All subjects were released at their site of capture after testing.

#### Materials

**Fig 2:**
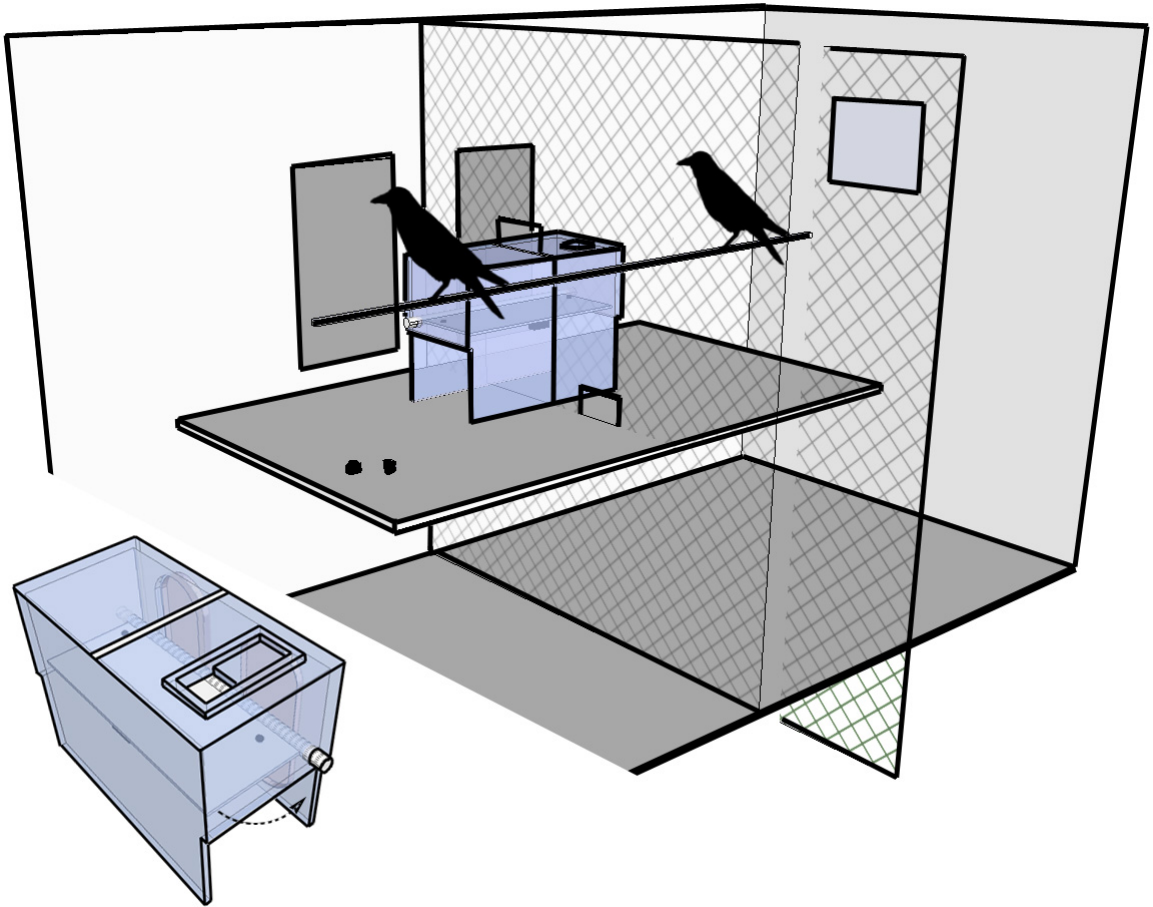
Experimental setup and apparatus used by group one. The diagram of the box shows the cooperative apparatus. Stones dropped through the hole on the surface of the box would hit a baited central platform, causing the platform to swing downwards and release the food. The large diagram shows the setup of the box between two cages. The crow on the left (‘passer’/subject) had to pick up a stone and place it next to one of two gaps in the wire mesh divider (passing locations). The crow on the right (‘dropper’/partner) could then drop the stone into the apparatus and release food into both cages. For group two, the hole in the apparatus was on the left.

The apparatus consisted of a clear acrylic box (LxWxH: 40x20x30 cm, Fig 2) with a collapsible middle platform which could be baited with food. Stones dropped through a 5cm^2^ hole on the top surface of the box would collapse the platform, allowing subjects to obtain the rewards. The apparatus was placed between two adjacent cages, separated by a wire mesh wall. The hole was only accessible to the bird on one side of the wire mesh divider; however collapsing the platform would provide rewards to both birds. A perch ran parallel to the apparatus providing the crows with full visibility of each other and of the rewards on either side of the apparatus.

#### Procedure

All subjects were habituated to the apparatus and the testing cages. They were then individually trained, from one side of the divider, to drop stones into the apparatus to collapse the platform. Birds initially nudged stones placed in close proximity to the hole, and training continued until they reliably picked stones up from the table and dropped them into the hole.

#### Test 1a: Spontaneous Stone Passing

Following training, birds were paired with another individual to take part in a spontaneous cooperation task. One bird was let into each cage: one with access to the hole (the ‘dropper’/partner), and one provided with a stone (the ‘passer’/subject). Two gaps in the dividing mesh wall acted as passing locations, one on top of the apparatus, and one along the table, where the passer bird could place the stone within reach of its partner. The partner could then drop the stone into the apparatus to collapse the platform, providing rewards to both birds. If the stone was not passed the dropper was unable to trigger the apparatus. Each pair received seven trials per block, consisting of 4 ‘cooperation’ trials (as described), and three ‘motivational’ trials where the experimenter placed the stone on the dropper’s side. A trial ended when the crows obtained the food, or when 2 minutes had elapsed. Testing continued until crows passed the stone on 75% of trials within one block.

#### Test 1b: Inequity Aversion

Birds then received an Inequity task, where the quantity of food available to each bird was varied on each trial. There were five types of trial: (1) *low equity* (both the passer and the dropper received one small meat reward), (2) *high equity* (both partners received one large meat reward), (3) *low inequity* (the passer received one small reward, whilst the dropper received five small rewards), (4) *high inequity* (the passer received one large reward, whilst the dropper received five large rewards) and (5) *super inequity* (the passer received one small reward, whilst the dropper received five large rewards). Each small meat reward was around 150 mm^3^, while each large one was 750 mm^3^. Birds received 5 blocks of 10 trials. Trials lasted 1 minute, and whether or not the target bird passed the stone within this time, was recorded.

### Group 2

#### Subjects

Subjects were 6 wild-caught New Caledonian crows, captured from two sites on Grande-Terre in April 2014 and housed in a 10-cage outdoor aviary. Crows were housed and fed as in Experiment 1. All crows were released at their site of capture at the end of testing.

#### Materials

The cooperative apparatus was identical to Experiment 1. In addition, an ‘individual’ apparatus was used in Test 2b, comprised of a smaller Perspex box (18 x 12 x 9 cm) with a collapsible middle platform. This apparatus could be operated individually by dropping a stone into a (5 cm^2^) tube on the top surface.

#### Procedure

All birds were individually trained to drop stones into the cooperative apparatus, as in Experiment 1. In preparation for Test 2b, they were also trained to attend to different food rewards placed inside the cooperative and individual apparatuses. During training, both apparatuses could be operated individually, and on each trial one apparatus contained meat (high value) and one contained dog-food (low value). A single stone was provided. Birds continued training until they dropped the stone into the apparatus containing meat on 9/10 trials, indicating that they attended to the contents of the two apparatuses before making a choice. To ensure birds would still work for dog-food after this experience they received 10 trials dropping stones into an apparatus containing only dog-food in an adjacent room.

#### Test 2a: Spontaneous Stone Passing

Following training, birds took part in a spontaneous cooperation experiment. The procedure was identical to Task 1a except that two, not three, motivational trials were given per block.

#### Test 2b: Social v Asocial Conditions

Birds then received a social vs. asocial test, where the presence or absence of a partner was manipulated across blocks of trials. Here, the cooperative and individual apparatus were both presented, 50cm apart, in the passer’s cage (see S1 Movie for set-up). The individual apparatus could be operated alone, but the cooperative apparatus required a partner. On all trials the cooperative apparatus contained meat (high value) and the individual apparatus contained dog-food (low value). A single stone was provided in between the two apparatuses. If individuals possessed cooperative cognition, they were expected to prefer to pass the stone on top of the cooperative apparatus in social trials, when their partner was present, to obtain the high value reward. However, they were expected to drop the stone into the individual apparatus, in asocial trials, to obtain the low value reward when obtaining the high value reward was impossible. Given the small sample size (n = 6), individual performance was analysed in addition to group means to determine whether any individual bird showed choices consistent with cooperative cognition.

If birds did not pass the stone or obtain either reward within two minutes the trial was ended. Birds received 5 blocks of 4 trials in each condition. Blocks were pseudorandomised, with the provision that all birds received a social block first and the same block was not repeated more than twice consecutively. Birds were given blocks of each condition, rather than individual trials to minimise the disturbance caused by moving the partner in and out of the adjacent cage, and to maintain their motivation for the task.

#### Test 2c: Inequity Aversion

To test the robustness of our findings with group 1, birds in group 2 received a different inequity task where the quality, rather than quantity of food was varied. Birds received four types of trial, pseudorandomised in four blocks of 10 trials, with the provision that trials were not repeated more than twice consecutively. Trial types were: (1) *low equity* (both partners received dog-food), (2) *high equity* (both partners received meat), (3) *advantageous inequity* (the passer received meat, but the dropper received dog-food), and (4) *disadvantageous inequity* (the passer received dog-food, whilst the dropper received meat). Only the cooperative apparatus was presented. Trials lasted 1 minute, and whether or not the bird passed the stone within this time was recorded.

#### Analysis

Experimental trials were video recorded for later analysis. A second observer coded 10% of the videos, finding 100% agreement with the data, Kappa = 1.0. Actions were coded as ‘stone passing’ when the subject placed the stone within reach of the partner, who then used it to trigger the apparatus. In trials where no partner was present stones were considered to have been ‘passed’ when they were placed within 5cm either side of the divider (unambiguous passing) or placed within the potential reach of their specific partner, determined by the pair’s behaviour on social trials (pair-specific passing).

Individual performance on the social vs. asocial task was assessed using binomial tests to determine whether the subject chose the cooperative apparatus more often than chance. Mixed logistic regressions, with random intercepts for birds, were used to analyse the group level performance on the social vs. asocial and inequity aversion tasks, and a GLMM assuming a negative binomial distribution was used to analyse the latency to pass the stone in the social vs. asocial task. Holm-Bonferroni corrections were applied to account for multiple tests. All analyses, except the binomial tests, were conducted using the glimmix procedure in SAS version 9.4, using the satterthwaite approximation for the denominator degrees of freedom. Binomial tests were conducted in Excel.

## Results

### Test 1a & 2a: Spontaneous Stone Passing

All birds began passing stones within 2–21 trials, and reached criterion with 8–24 trials (Table 1). The first group of crows took 5.50 ± 1.57 trials (mean ± s.e.m.) to start passing the stones, and the second group took 9.67 ± 2.94 trials (mean ± s.e.m.). Eight of the twelve crows were naïve to stone passing, as they had not performed the role of a dropper before the test. Naïve crows took 8.13 ± 2.45 trials (mean ± s.e.m.) to pass stones; while crows with experience of dropping took 6.50 ± 1.85 trials (mean ± s.e.m.). In group 2 all passes were made on top of the apparatus, not along the table.

**Table 1:** Summary of pairs and their performance on the spontaneous stone passing task (Test 1a & 2a).

Group	Subject	Partner	Trial to start passing	Trial to reach criterion	Naïve to stone passing?	Related to partner?
1	BBW	RGR	2	8	Yes	No
1	OBB	RGR	9	20	Yes	No
1	RGR	BBW	9	24	No	No
1	YBB	GBG	2	8	No	Yes
1	GBG	YBB	2	12	Yes	Yes
1	OGG	YBB	9	16	Yes	No
2	D4-R	LB	5	8	Yes	Yes
2	RW-Y	D3-R	21	24	Yes	No
2	D4-B	D4-R	15	24	Yes	Yes
2	LB	D4-R	5	8	No	Yes
2	D3-R	D4-R	10	16	No	No
2	D3-B	D4-R	2	16	Yes	No

*Note*: Eight subjects were naïve to stone passing as they had not performed the role of ‘dropper’ before their test. Six of the pairs were likely to be related (subjects were caught together), and six were likely to be unrelated (caught at different sites, at different times). Criterion: 3 out of 4 stones passed in one block.

### Test 2b: Social v Asocial Conditions

**Fig 3:**
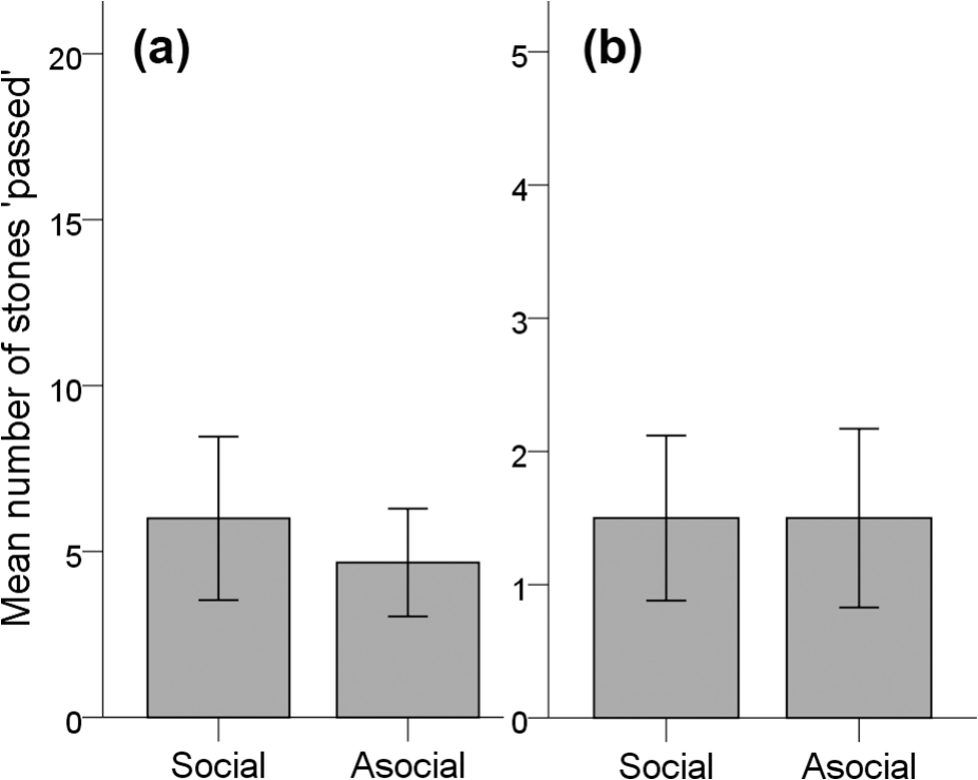
a & b. Mean number of stones passed on the cooperative apparatus in the social and asocial conditions. (a) total, 20 trials (b) first trial in each social or asocial block, 5 trials. Error bars ± S.E.

If subjects possessed cooperative cognition they were expected to significantly prefer to pass the stone on social trials, but prefer to drop the stone into the individual apparatus on asocial trials (≥15/20, binomial tests: *p* < .05). However, individually, none of the 6 birds tested showed this pattern (Table 2). At the group level, a mixed logistic regression was performed on the likelihood of stone passing on all trials in the social and asocial conditions (6 birds, 240 observations). The between bird variance was 2.98. The logistic regression did not find condition (social or asocial) to be significantly related to the probability of passing a stone (Type 3 test: F(1, 238) = 1.78, *p* = .18). In total, birds passed a stone on 6.00 ± 2.46 trials (mean ± s.e.m.) in the social condition and 4.67 ± 1.62 trials (mean ± s.e.m.) in the asocial condition (Fig 3A.). Furthermore, on their first trial of each block, birds passed the stone exactly as often in the social and asocial conditions (Fig 3B).

**Table 2:** Individual performance in the social and asocial conditions (Test 2b).

Bird	Total /20		1^st^ trial per block /5	
	Social	Asocial	Social	Asocial
D4-R	4*	**3** *	1	1
RW-Y	11	9	2	3
D4-B	0*	**0** *	0	0
LB	0*	**2** *	0	0
D3-R	6	**4** *	2	1
D3-B	**15** *	10	4	4
Mean:	6.00	4.67	1.5	1.5

*Note*: scores indicate the number of trials on which subjects passed the stone on top of the cooperative apparatus, ≥15/20 = significant preference for the cooperative apparatus, ≤5/20 = significant preference for the individual apparatus. Results are given for the total number of trials in each condition, and for the first trial of each block (5 trials per condition).

*binomial test, *p* < .05.

Bold: subject made the optimal choice for that condition.

Considering only those trials where stone passing occurred, we found no evidence that birds took more time to pass the stone in asocial trials compared to social trials. A GLMM assuming a negative binomial distribution, with random intercepts for birds, was performed on the time taken to pass the stone in the social and asocial conditions, using only the trials when stone passing occurred (6 birds, mean number of observations per bird = 15). The between-bird variance was 0.136. Here, no significant effect of condition was found on time taken to pass the stone (Type 3 test: F(1,55) = 1.29, *p* = 0.26).

Birds either passed the stone or obtained the individual reward within 2 minutes in all except 4 trials (all RW-Y, social condition). In the asocial condition passing was unambiguous in 21/26 trials and stones were placed within extended reach of their partner on the remaining 5 trials (RWY: 4; D4-R: 1).

### Test 1b & 2c: Inequity Aversion

For the inequity aversion task with group one a mixed logistic regression was performed on the probability of passing the stone in the five conditions these birds experienced: low equity, high equity, low inequity, high inequity and super inequity (6 birds, 300 observations). The between-bird variance was 2.01. Here, condition was found to be significantly related to the probability of passing the stone (Type 3 test: F(4, 295) = 6.15, *p* < .001). Specifically, the low equity condition differed significantly from three of the four other conditions. Compared to the low equity condition, the odds of passing were 6.09 times higher under the high equity condition (*p* < .001). However, they were also 7.2 times higher under both the high inequity and super inequity conditions (both *p* < .001). The odds of passing were 2.81 times higher under the low inequity condition, but this difference was not significant against a Holm-Bonferroni corrected alpha of .007 (*p* = .021). Thus, birds passed more often in the two conditions which had the highest levels of inequity, and the high equity condition, than they did in the low equity condition. There were no other significant differences between conditions (pairwise comparisons: Table 3).

**Table 3:** Pairwise comparisons from the logistic model of the inequity conditions experienced by group one (Test 1b).

									95% Confidence Int-erval for Odds Ratio	
Comparison			Differences on the Log Odds Scale	Odds ratio	Standard Error	DF	t value	p value	Lower	Upper
Super Inequity	vs	High Inequity	0.00	1.00	0.59	295	0.00	1.000	0.31	3.22
Super Inequity	vs	Low Inequity	0.94	2.56	0.54	295	1.76	0.080	0.89	7.35
Super Inequity	vs	High Equity	0.17	1.18	0.58	295	0.29	0.773	0.38	3.70
Super Inequity	vs	Low Equity***	1.97	7.20	0.52	295	3.8	**>0.001**	2.59	20.03
High Inequity	vs	Low Inequity	0.94	2.56	0.54	295	1.76	0.080	0.89	7.35
High Inequity	vs	High Equity	0.17	1.18	0.58	295	0.29	0.773	0.38	3.70
High Inequity	vs	Low Equity***	1.97	7.20	0.52	295	3.8	**>0.001**	2.59	20.03
Low Inequity	vs	High Equity	-0.77	0.46	0.52	295	-1.49	0.137	0.17	1.28
Low Inequity	vs	Low Equity	1.03	2.81	0.44	295	2.32	0.021	1.17	6.73
High Equity	vs	Low Equity***	1.81	6.09	0.50	295	3.6	**>0.001**	2.27	16.35

Note:

***p < .001

**Fig 4:**
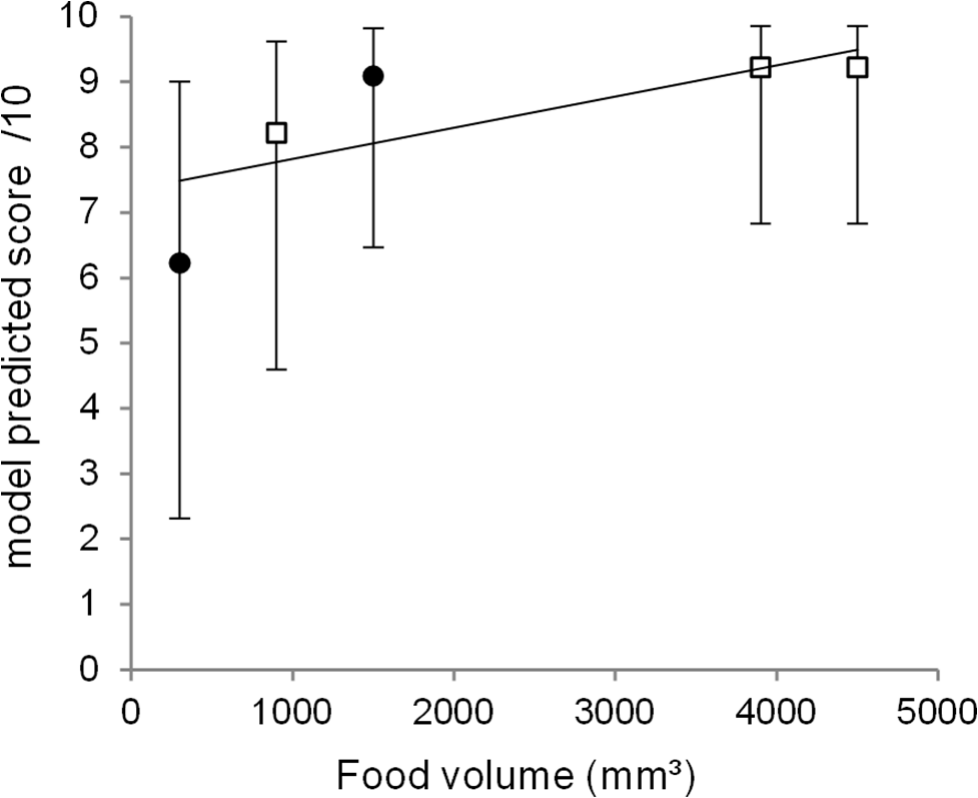
Model-predicted probability of stone passing in relation to the total volume of food in the apparatus (Test 1b). Crows increased their passing rate as food volume increased. Circles = equity conditions, squares = inequity conditions. Error bars = 95% confidence intervals.

Our results indicated that birds passed the stone least often when the total amount of meat provided in the apparatus was at its lowest. Thus, considering meat volume as a continuous variable, we tested for a linear trend in stone passing with increasing meat volume, which was found to be significant (F(1,798) = 15.14, *p* < .001). Each increase in meat volume of 1000mm³ was associated with an increase in the odds of passing the stone by a factor of 1.51. The fitted trend can be seen in Fig 4. Although we saw from the plot that a linear trend was not the best possible fit, we had insufficient unique values to fit a quadratic curve to our data. Finally, there was no indication that birds began passing less in the Inequity conditions over the course of the experiment. Taken together, birds passed the stone exactly the same number of times in the first 5 and the last 5 trials of the three Inequity conditions.

**Fig 5:**
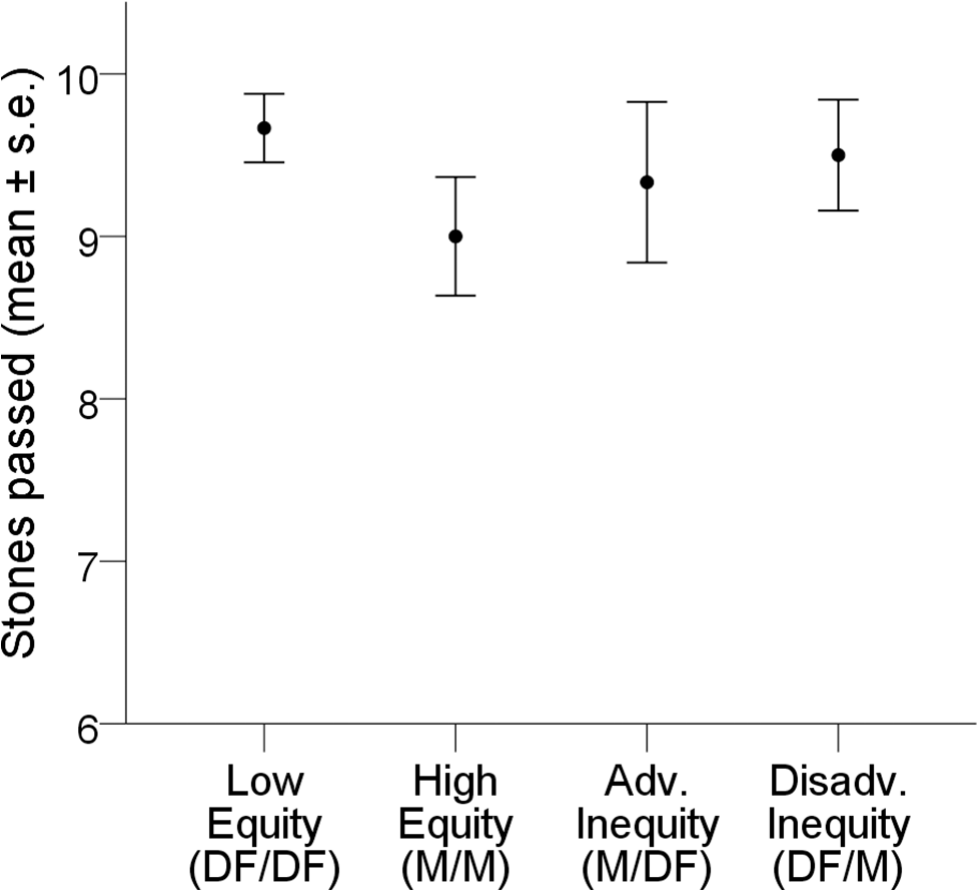
Mean number of stones passed in an inequity test by group 2 (Test 2c). Disadv. = Disadvantageous, Adv. = Advantageous, DF = dog-food, M = meat.

For group two, a mixed logistic regression was conducted on the probability of stone passing in the four inequity conditions experienced by this group: high equity, low equity, disadvantageous inequity and advantageous inequity (6 birds, 240 observations). The between-bird variance was 0.66. The inequity condition variable was not found to be significantly related to the probability of passing a stone (Type 3 test: F(3,236) = 0.43, *p* = 0.73). Birds were highly likely to pass the stone to their partner across all four types of trial (Fig 5, S1 Data).

## Discussion

Collaboration is considered to be the most complex form of cooperation, as individuals have to assume different but complementary roles [70]. The behaviour of the NC crows studied here fits this definition. The subject picked up a stone and placed it within reach of another crow, which then dropped the stone into a hole in an apparatus to collapse a baited platform. Each individual performed a different behaviour that enabled both subjects to achieve a shared goal, identical to that used in other studies on animal cooperation, namely, two rewards separated spatially on a platform [2,3,7]. Our results therefore show NC crows can spontaneously solve a complex cooperative task.

However, we found no evidence that their solution was based on an *understanding* of cooperation. The crows did not adjust their behaviour according to the presence or absence of a partner. They were equally as likely to pass the stone when there was no bird there to receive it, as they were when a partner was available to take the stone and use it to collapse the platform. Strikingly, on their first trial of each social and asocial block–before they could receive feedback about whether or not their actions led to reward–NC crows passed the stone on *exactly* the same number of trials in the two conditions. Individually, none of the six birds showed a pattern of behaviour expected from an individual possessing cooperative cognition: a preference for the cooperative apparatus in the social condition and a preference for the individual apparatus in the asocial condition. NC crows also demonstrated no sensitivity to inequity. In group one subjects were significantly less likely to pass the stone in the Low Equity condition (where both birds received one small piece of meat) than in three of the four other conditions, including those with the highest levels of inequity. Thus, passing was related to the total volume of food in the apparatus, but not to the level of inequity. Subjects passed stones even when they received 25 times less food than their partner, and after repeated exposure to various inequity conditions. No inequity aversion was found when the quantity (group 1) or the quality (group 2) of the rewards was varied, despite the task meeting the optimal conditions for inequity aversion: subjects were caged close together, side-by-side, and had to perform a task to obtain the rewards [15].

Our results are based on a small sample (two groups, each of six birds), thus it remains plausible that in a larger group we might find individual birds that do respond to their partner’s presence and absence, or show sensitivity to inequity. Our group-level analyses, based on these small samples, should be interpreted with some caution. However, as they stand, our results are a prime example of how apparently complex cooperative behavior can be underpinned by simple cognitive mechanisms. While NC crows produced behaviour that could be described as collaborative, this performance was not underpinned by an understanding of collaborative action, in terms of the roles each crow took and the rewards they gained for their behaviour. In the current study, it is likely the crows’ previous learning history led to their stone passing behaviors. During training, crows had been rewarded for picking up stones and dropping them into the hole. Thus, we suggest that the crows attempted to reproduce this behavior in the cooperative task, by attempting to place the stone as close to the hole as they could. Because the point closest to the hole was the communal area on top of the apparatus, crows placed the stones here, which resulted in the stone being within reach of the other bird. In support of this, crows in group two never passed stones to their partner through a gap in the divider on the table, even when the stone had fallen to the table and was closest to this potential passing location.

It is conceivable that complex social cognition possessed by NC crows in our study was masked by problems with inhibition. NC crows may have recognized that there was no partner present or that the reward available to them was unequal, but they still failed to inhibit passing the stone. However, there are compelling reasons to reject this as a full explanation for our results. First, during training, subjects in group two were given experience choosing between the cooperative and individual apparatus when they were baited with different foods (high-value meat, or low-value dog-food), until they selected the apparatus containing meat on 9/10 trials. Therefore, before the experiment began, subjects were capable of switching their responses on each trial, by assessing the contents of two apparatuses before making a choice. Thus, it is likely that if the birds knew they needed a partner to operate the cooperative apparatus, they would also have been able to switch their choices according to the presence or absence of a partner. Furthermore, in the inequity aversion task, when only one apparatus was present, crows did not appear to fail solely because of an inability to inhibit actions. The crows in group one appeared to inhibit stone passing when the total amount of food in the apparatus was very low (Fig 4), but they did not do the same when the total available to *them* was very low, despite the fact that they repeatedly experienced these conditions of inequity.

An alternative possibility is that the crows’ *initial* solutions resulted from cooperative cognition, but their performance on the later tasks, such as the social vs. asocial task, was limited by behavioural momentum: later actions became guided by past reinforcement, overshadowing their cognitive abilities. While this scenario is possible, we do not think it is particularly likely. NC crows chose the cooperative apparatus on only 30% of trials in the social condition–despite being consistently reinforced for choosing this apparatus when their partner was present–and, overall, they preferred the low-value but consistent reward from the individual apparatus in both the social and asocial conditions. This suggests that birds did respond to reinforcement, but did not recognize that the *presence* of a partner determined whether they would be rewarded. It is therefore unlikely that they recognized the need for a partner in their initial solution of the task. Thus, it appears that crows did not fail the asocial and inequity conditions because they were simply unable to inhibit passing the stone or were demonstrating behavioural momentum. Instead, their behaviour strongly suggests that these NC crows did not understand that they needed another individual to act as a social tool, and they were not sensitive to inequity.

Our results place NC crows alongside orangutans, squirrel monkeys, owl monkeys, marmosets and tamarins [23,26–29], as species who fail to show sensitivity to inequity. They contrast with a recent study which found a trend for carrion crows and ravens to exchange a token for a low quality reward more often when their partner also received a low quality reward (equity), than when their partner received a higher quality reward (inequity) [25]. However, this evidence for inequity aversion in corvids is tentative at best. There was no difference in exchange rates between the inequity condition and a condition where subjects received the low quality reward with no partner present, indicating that they could be responding to reward type more than inequity. Furthermore, when the subject received no reward at all for their actions, they were equally likely to exchange the token regardless of whether (a) there was no partner present, (b) their partner also received no reward (equity) or (c) their partner *did* receive a reward (inequity). Whether or not inequity aversion is a feature in the corvid lineage is, therefore, presently unclear.

The performance of NC crows is similar to that of rooks when faced with a cooperative task. Seed et al [7] found that rooks were able to pull two strings simultaneously to bring a baited platform within reach. However, if their partner was delayed, the rooks (unlike chimpanzees, elephants, or dogs [2,3,9]) were unable to wait for their partner to enter the testing room before pulling the string. Seed concluded that rooks did not understand the role their partner played in this task–or at the very least any understanding was masked by problems with inhibition. Thus, the rooks’ behaviour appears to be similar to that of the NC crows tested here. Similarly, Scheid and Noë [71]–testing a different population of rooks–found no evidence that rooks would delay pulling if their partner was not present at the apparatus (rooks pulled on 21/26 of such trials). However, as partner arrival time was not experimentally manipulated, it is unclear whether or not these birds understood the role of their partner in this case.

The results reported here provide insight into the selective pressures that lead to the evolution of cooperative cognition. Small family groups [46], mobbing (Fig 1), high tolerance for conspecifics [72,73], tool use [52], tool manufacture [52,54,55], metatool use [56,57], context-dependent tool use [59], an abstract understanding of tool properties [57,58] and sophisticated causal or inferential reasoning [60,61,64,74] do not appear to be sufficient for the evolution of the cognitive mechanisms involved in producing and maintaining cooperation between unrelated individuals. Thus, the evolution of an ability to understand that conspecifics can be used as a social tool, seen in humans, certain primates, elephants and perhaps dogs, seems unlikely to have been solely driven by the cognitive abilities associated with tool use and tool manufacture. It is more likely that aspects of these species’ social environments drove the evolution of cooperative cognition. Furthermore, complex social cognition does not appear to be a prerequisite for the evolution of the cognition involved in producing complex tool behaviors and making causal inferences. Based on the NC crows’ poor performance on the social task demonstrated here, it seems highly unlikely that the technical skills of NC crows are a byproduct (exaptation) of complex social cognitive mechanisms.

Our results on inequity aversion are in line with the hypothesis that responses to inequity evolve in order to facilitate frequent cooperation between non-kin [1,42,43]. Although NC crows perform some cooperative behaviours in the wild (i.e. mobbing, Fig 1), they have low levels of sociality and are less cooperative than chimpanzees or capuchins [75]. Thus, the finding that NC crows’ combination of enhanced physical cognition, moderate cooperative behaviours and low sociality has not led to inequity aversion in this species, fits with the theory that inequity aversion evolves as a mechanism for cooperative, social individuals to engage in optimal partnerships and abandon inequitable ones [43–45].

Finally, our results support the view that at least some aspects of intelligence evolve in a domain-specific manner. It has recently been suggested that human cognition evolved with a domain-general form [76–83], and so uses “a common set of computations to process information from a broad range of technical and social domains” [76]. However, the cognitive mechanisms underpinning complex tool behaviors and sophisticated causal reasoning in NC crows do not allow this species to understand that a conspecific can act as a social tool, nor to track inequity, during cooperation. Thus, complex cognitive mechanisms can evolve that are unable to process both technical and social information. While it seems highly likely that a positive feedback loop existed between technical intelligence and social intelligence during human cooperative hunting [84], at least in NC crows, aspects of these two cognitive spheres are decoupled.

## Supporting Information

S1 Movie Example videos of the spontaneous stone-passing task and the social vs. asocial conditions (Tests 1a & 2b). (MP4)

S1 Data Individual performance in the inequity conditions (Tests 1b & 2c). (XLSX)
